# Late Metastatic Melanoma after 25 Years: A Case Report and a Brief Literature Review

**DOI:** 10.1155/2020/2938236

**Published:** 2020-10-30

**Authors:** Elena Pescarini, Gabriela Spanikova, Zacharia Mbaidjol, Eleonora De Antoni, Vincenzo Vindigni, Franco Bassetto

**Affiliations:** ^1^Clinic of Plastic and Reconstructive Surgery, Padova University-Hospital, Italy; ^2^Clinic of Paediatric Surgery, Jessenius Faculty of Medicine, Comenius University in Bratislava, Slovakia; ^3^Plastic and Reconstructive Surgery, Queen Victoria Hospital, East Grinstead, UK

## Abstract

The incidence of cutaneous malignant melanoma has shown a drastic increase over recent decades, and approximately 70% of newly diagnosed melanoma are tumors with a Breslow thickness less or equal to 1 mm. In the literature, there are well-documented rare cases of late metastasis of thin melanoma, and given their growing incidence, it is expected in the future to see more cases of late recurrence. We present a case report of a metastatic cutaneous melanoma 25 years from diagnosis and a review of the literature. A 61-year-old female patient presented with a newly discovered asymptomatic nodule on her thigh. Her relevant past medical history included a completely excided lesion with Breslow 1.4 mm thickness in 1989 for which she was followed up according to the guidelines and subsequently declared cured after 10 years of surveillance. Fine-needle aspiration and cytological analysis of the lesion proved to be a subcutaneous localization of malignant melanoma. The lesion was completely excised, and the patient has remained disease free since her surgery. The aim of this case report is to emphasize that late metastasis remains uncommon but a definitive cure from melanoma cannot always be considered a disease-free interval of 10 years. Physicians should always be aware of previous melanoma diagnosis with newly discovered suspicious lesions. Better patient education could improve the detection of recurrence and secondary melanomas without any need for more frequent follow-up visits and a prolonged follow-up time.

## 1. Introduction

Melanocytes are the normal pigment-producing cells in the skin and their malignant proliferation results in melanoma. Cutaneous melanoma is one of the most common types of neoplasia in young people and has a high mortality rate in this population. Melanoma is a public health issue due to its growing incidence and frequency of diagnosis in people under 59 years of age and the healthcare costs and mortality associated with it. The prevalence of melanoma is 9 cases in 100,000 people with a mortality rate of 2.3 cases per 100,000. This increases with latitude and is closely related to skin type, showing a huge binding with prototype I-II. Even though the incidence is increasing, the mortality rate seems to be stable [1]. Primary prevention of melanoma is important so that definitive treatment can occur in early stages and to prevent metastasis from occurring. Surgery remains the gold standard treatment for localized cutaneous melanoma. Once metastasis has occurred, melanoma has a poor prognosis due to lack of effective treatment. The overall survival (OS) is connected mostly with the site of recurrence. Surgery for metastatic disease can also affect prognosis in addition to being a palliative therapy [2, 3]. For this reason, the surveillance of patients is an important tool to check the progression of the disease.

In the literature, there are case series that show late recurrence rate of melanoma ranging from 0.98% to 6.7% [2, 4]. One study of 1372 10-year disease-free patients demonstrated a relapse in 77 patients (5.6%). Characteristics found to be connected with an increased risk of late recurrence include Breslow thickness > 2 mm, age under 40 years, and Clark level IV/V [5].

We present a case of late melanoma recurrence 25 years after the excision of the lesion, presenting with an in-transit metastasis in the thigh, and a brief literature review.

## 2. Case Presentation

A 61-year-old woman presented to our outpatient clinic complaining of an indolent, firm, hard subcutaneous nodule on her left thigh. She believed that she had a sebaceous cyst as she had an abscess on the contralateral thigh few years previously. In addition to the removal of a malignant melanoma 25 years ago, her past surgical history included a hysteroadnexectomy for endometriosis and a drainage of a suppurating sebaceous cyst on the right thigh. She also suffered from endometriosis, abdominal chronic pain, and chronic constipation.

In 1989, she was diagnosed with malignant melanoma, which was located on the middle third of her left calf. It was excised with a wide margin and the pathological report from the excisional biopsy revealed a “superficial spreading melanoma with epithelioid cellular morphology, median mitotic rate, low inflammatory infiltrate throughout the tumor or along it, Clark level III, and Breslow 1.4 mm.”

At the time of the excision, there was no evidence of distant metastasis or pathological regional lymph node spread, and no other atypical melanocytic lesions were observed. Subsequently, the scar was excised with an additional margin and no residual tumor cells where found. No further therapy was planned, and she underwent regular follow-up for 10 years without any signs of relapse and no other cutaneous neoplasms were noted during physical examination. The treatment she underwent was in line with guidelines at that time as it was a complete excision with negative histopathological margins [6].

The characteristics of our patient's primary melanoma were similar to those related to lesions with late metastasis. She was a female, with an extremity located superficial spreading melanoma and intermediate (1.0 to 3.0 mm) thickness, Clark's level III. She presented with Stage I disease (in the pathological report there was no sign of ulceration), and the recurrence was regional [4]. A secondary melanoma manifesting as a confined subcutaneous lesion was highly unlikely given their extremely low prevalence and poor prognosis [7].

A fine-needle aspiration of the newly discovered lesion was promptly performed, and the cytological examination revealed a malignant tumor compatible with a subcutaneous localization of a malignant melanoma. Immunohistochemistry findings demonstrated positive staining for Melan A and HMB 45. A complete excision of the lesion with a narrow margin was performed in accordance with the guidelines.

The final pathological reports described a “subcutaneous localization of melanoma, with necrotic areas, a mitotic index of 15/mm^2^, and an angiolymphatic invasion”; the immunochemistry staining showed HMB 45+, Mel ARED+, and MIB 1 40%. The immunohistochemical profile along with the morphological appearance agreed with the diagnosis of metastatic melanoma. Furthermore, the genetic analysis reported a BRAF codon 600 mutation in exon 15.

Thorough investigation, including a total-body PET-CT, failed to reveal any other primary melanoma or metastasis. For our patient, we discussed the choice of not performing SLN biopsy in our multidisciplinary tumor board and subsequently scheduled nevus mapping and radiological exams.

The patient was referred to an oncologist and had yearly clinical and dermatoscopic follow-ups. The latest was in 2019. She was not started on any adjuvant therapy.

As an incidental finding, a contrast enhancement of the right superior lobe of her thyroid was also discovered during the staging CT for which she was referred to her general practitioner. She has until now not shown any recurrence of her disease.

## 3. Discussion

Despite an increased level of public health education and awareness about melanoma, the mortality is greater than that caused by all other types of skin cancers [8].

The mortality is strictly related to the stage of the disease with metastatic recurrence occurring in about 65-81% of patients in the first 3 years after excision [9]. Though the tumor thickness is a prognosis indicator, it is not an absolute predictor of the length of disease-free interval. Local lymphadenopathy and local skin metastasis are superior survival indicators than distant metastasis [10].

Early metastasis, that is, 3 years after the diagnosis, is associated with lesions with ulceration, increased thickness, and with patients with an history of nonmelanoma skin cancer (NMSC).

The potential and pattern of metastasis for melanoma are associated with tumor characteristics, tumor cell extrinsic factors, tumor macroenvironment, and tumor microenvironment. These can have very variable and powerful effects on tumor cells, both inhibiting and promoting metastastasis [11, 12].

Most of the national guidelines recommend a 10-year follow-up as after this protracted disease-free time period patients are frequently considered “cured,” and metastasis is most commonly seen in the first years following the excision.

Melanomas have however a capricious clinical course and late recurrence defined as metastasis after 10 years from initial treatment have also been reported. This represents an uncommon event but is nonetheless a disturbing characteristic of the disease. The AIOM (Italian Oncologist association) underlines that in a 10-year interval it is possible to find 229 recurrences and 61 new primary melanomas every 1000 patients examined. Most melanomas metastasize within 5 years after the primary tumor has been removed but late metastasis has been reported [4, 13].

The term late metastasis is used to indicate a distant recurrence of a tumor after a period exceeding 10 years from the final treatment of the primary tumor and without evidence of recurrence at the site of the primary tumor [14, 15]. The first case of late metastasis was described in 1931 by Wilbur [16].

In the literature, it is possible to find several case reports and case series regarding late metastasis (Table 1), but it is seldom possible to find patients with metastasis from a cutaneous melanoma after 20 years from the definitive treatment. Moreover, some series in the literature lack information regarding both the tumor histology and the patient's clinical characteristics (Table 2).

Crowley and Seigler reported an incidence of 2.4% of late metastasis in 168 patients [4] and Briele et al. reported a 6.7% incidence, that is, 7 patients, in his series of 105 cases [14]. Callaway and Briggs described five patients with late metastasization accounting for a 0.98% incidence in a series of 536 patients [7]. Shaw et al. reported an incidence of 2.7% patients (34 patients affected with late metastasis) in their series of 1283 patients [17].

Classically, metastasis is thought to occur late in tumor development following tumor angiogenesis and lymphatic invasion, allowing distant spread of malignant cells. For this reason, the progression of melanoma may be more complex than the one previously believed. Some melanoma cells may spread much earlier despite no clinically apparent metastasis adding complexity to this process. This suggests the presence of some barriers to overcome to progress to macrometastatization and introduces the concept of dormancy [18].

The Australian pathologist Rupert Willis first used the term dormant to refer to an arrest in the cell cycle or a nonproliferation state that results in a prolonged G_0_ phase. Late recurrence phenomenon has been described through the dormancy theory as “a period in cancer progression in which residual disease is present but remains asymptomatic” [5].

Dormancy also refers to the period between the primary excision and the subsequent metastatic recurrence [11]. Even decades after the primary treatment, these dormant malignant cells can awaken and cause recurrent diseases. This is sometimes called the parallel progression model of metastasis [11].

The mechanisms regulating this phenomenon are poorly understood but the literature suggests the presence of intrinsic factors (growth arrest in a prolonged G0 phase or inability to recruit new blood vessels and therefore to spread) or the presence of an immunosurveillance that actively constrain the growth of the metastasis. This concept is still highly debated. It is believed that changes in the immune system might be connected with the occurrence of late metastasis [19]. In an uveal melanoma mouse model, Eyles and colleagues found the first single tumor cell to spread less than 3 weeks after the clinical onset of the primary tumor which demonstrated that immunosurveillance controls the growth of primary melanoma and check the metastatic outgrowth [20]. This theory was highlighted by the inadvertent transmission of melanoma through an organ donor to an organ recipient [10]. Furthermore, the fact that late metastasis bears the genetic signature of early disseminated tumor cell could be a proof of immunosurveillance-induced tumor dormancy [18].

Furthermore, tumor cells could be capable of pausing their expansion in a hostile environment due to a mismatch interaction between the tumor cell and the extracellular matrix. A change in the cellular environment is considered to be connected with tumor dormancy and is believed to be the key to the transition between micrometastasis to macrometastasis, which can happen several years after the tumor eradication [11].

Moreover, metastasis can seed additional tumor cells and create new metastasis [11]. It is worth to mention that some authors state that these “late” recurrences can be classified as “melanoma of unknown primary (MUP) [21].” When metastasis is clinically evident in a patient, the prognosis is poor.

In our case, we did not consider the lesion to be a MUP as it did not fit into the original definition published in 1963 by Dasgupta et al. [22, 23]. A second melanoma was also highly unlikely due to the negative clinical examination and PET study performed. Moreover, during a 5-year follow-up, no second melanoma or malignancy as well as no evolution of the clinical scenario was found.

There are many studies evaluating the risk factors connected with the likelihood of metastasis for melanoma patients [24–26]; sadly, there are no consensus regarding the risk of metastasis, especially for late metastasis, [27] and there are only a limited number of studies reporting on patients with late melanoma recurrence due to the low frequency of this phenomenon.

A study of 168 patients presenting with late metastasis could not identify specific risk factors [4].

For this reason, it is extremely hard to determine which patients will need to undergo long-lasting follow-ups. It is worth mentioning that a study by Callaway and Briggs inferred that there was an absolute dominance of female patients with extremity melanoma in the group of patients with disease-free intervals longer than 25 years [7].

In the literature, patients with early metastasization show a high percentage of in-transit or regional metastases as opposed to distant metastases which are more likely to occur with late metastasization [24]. In a study analyzing 1881 melanoma patients for more than 10 years, Hansel et al. did not identify any significant risk factor for late metastasization [28]. Additionally, it appears that most of the malignancies are already disseminated when the primary tumor is detected [29] and that late metastasization is strictly bound to the fact that tumor cells can enter a temporary mitotic arrest or dormant state.

The late metastasis found in our patient fulfills more the criteria of the “in-transit metastasis” as it was a subcutaneous deposit of tumor cells between the primary melanoma and the regional lymph nodes. The American Joint Committee on Cancer (AJCC) terms in-transit metastases as any subcutaneous or skin metastases that are found more than 2 cm from the primary tumor but before the regional nodal basin. The survival connected with in-transit metastases is quite poor with a 5-year survival rate of 25% and almost no 10-year survival. In fact, these are a manifestation of an advanced disease and the TNM staging system assigns to in-transit metastases a separate N2c when they arise in absence of nodal metastasis; our patient could therefore be classified at Stage III. In our case, the in-transit metastasis appeared as a firm subcutaneous nodule around 1.5 cm in size. Usually, these lesions are erythematous pigmented or nodular and rarely appear as a flat plaque.

In some cases, sentinel lymph node (SLN) biopsy can be performed by injecting radiocolloid and/or blue dye around the most proximal in-transit lesion [30]. The likelihood of nonclinical evident metastatic lymph nodes is elevated when an in-transit metastasis is found after at least a disease-free survival (DFS) period of 6-12 months [31]. For this reason, the possibility for a SLN biopsy should always be discussed. In our case, it was not performed after discussion in our MDT as this was in line with the Italian melanoma guidelines at that time.

Therefore, it is of paramount importance to schedule outpatient surgical, dermatological and oncologic follow-ups. The patient should undergo an annual full skin assessment, with a physical examination that includes the regional lymph nodes palpation. This needs to be done keeping in mind that most of the recurrence are detected by the patient himself [32].

Furthermore, detection of early local or regional recurrence will determine the possibility of surgical resection and can allow to potentially identify a second primary tumor and early distant recurrences. In fact, incidence of a second melanoma is increased in melanoma patient with a cumulative risk swinging from 2 to 5 percent at periods from 2 to 5 years after the first melanoma has been discovered [2, 33].

There is no evidence follow-ups can change favorably the prognosis of a treated patient, nevertheless AIOM suggests a 10-year follow-up for all melanoma patients with outpatient clinic appointments and radiological staging exams. It is also encouraged that a patient with increased risk factors such as fair skin prototype, positive family history, and dysplastic nevi syndrome should be monitored. All patients should also be educated regarding the increased risk of secondary melanoma, the need of photoprotection, and how to perform a cutaneous autoexam to find early recurrence or neoplasm. The GP should also be aware of a lifetime increase risk of new primaries and recurrence. An annual dermatological mapping of the nevi should be performed lifelong [34]. Moreover, the involved team should inform patients to be in contact with their GP following the conclusion of the oncology outpatient follow-ups.

## 4. Conclusion

Definitive cure from melanoma cannot always be considered a disease-free interval of 10 years. Physicians should always be aware of a previous diagnosis of melanoma. Improved understanding of the characteristics of the disease and the concept of tumor dormancy will help define which lesions are prone to late recurrence and metastasis. This in turn will allow to develop new therapies. Follow-ups and patient awareness of the risk of recurrence are the main tools that physicians can use to uncover new metastatic disease and are of paramount importance because a prompt diagnosis can give the possibility to start an aggressive chemosurgical therapy.

## Figures and Tables

**Table 1:** List of publications regarding the frequency of late metastases (usually >10 years) from CMM (based on Farie et al.).

Source	Year	Total number of patients with MM	Late recurrence	Percentage	Median tumor thickness
Osella-Abate et al. [5]	2014	1372	77	5.6	NA
Faries et al. [35]	2013	4731	327	6.9	1.23
Brauer et al. [24]	2010	549	70	12.75	1.48
Hansel et al. [28]	2010	1881	19	1.01	2.24
Leman and Mac Kie [36]	2003	3822	25	0.65	2.21
Schmidt-Wendtner, et al. [37]	2000	1026	31	3.02	1.42
Shen et al. [38]	2000	1907	217	11.37	1.3
Peters et al. [39]	1997	1015	36	3.54	1.78
Tsao et al. [15]	1997	1010	20	1.98	1.38
Pearlman et al. [40]	1992	NA	19	NA	NA
Tahery and Moy [41]	1992	956	8	0.83	1.85
Levy et al. [42]	1991	NA	5	NA	1.03
Crowley and Seigler [4]	1990	651	168	25.8	1.6
McEwan et al. [43]	1990	264	11	4.16	1.31
Callaway and Briggs [7]	1989	NA	5	NA	1.8
Gutman et al. [44]	1989	94	6	6.38	NA
Landthaler et al. [45]	1989	NA	10	NA	1.71
Shaw et al. [17]	1985	1283	34	2.65	2
Briele et al. [14]	1983	105	7	6.66	1.91
Wilbur [16]	1931	NA	3	NA	NA

**Table 2:** List of case reports found in literature with information regarding the year and site of metastasis and the pathological characteristics of the primary tumor (usually >10 years) from CMM.

Source	Patient age	Years of late metastasis	Primary cutaneous melanoma	Metastasis location	Pathological classification of primary
Bouffard et al. [46]	65	27	Halo giant congenital nevus	Lungs	Not available
Karpathiou et al. [47]	83	40	Dorsal melanoma	Main stem bronchus	Not available
Goad et al. [48]	35	11	Right should7er	Multiple liver nodules, small bowel mass	Not available
Miller et al. [49]	70	30	Left thorax	Left thorax	Superficial spreading malignant melanoma, Breslow depth 2 mm, Clark level 4
Bredgaard and Lock-Andersen [50]	77, 56, 61	21, 25, 29	Thorax, upper back	Axilla, cubital fossa, left groin	Nodular malignant melanoma, Breslow depth 1.25 mm, Clark level III without ulceration; nodular malignant melanoma, Clark level III, no histological information
Saleh and Peach [51]	84	40	Left leg	Left calf and thigh	Epidermotropic metastatic melanomas
Vukomanovic et al. [52]	59	7	Giant pigmented nevus of left arm	Omentum	Breslow depth 0.78 mm, Clark level III
Gowrinath and Geetha [53]	65	10	Plantar malignant melanoma	Lungs	Not available
Terhorst et al. [54]	83	42	Back	Peritoneal seeding, greater omentum, appendix	Not available
Goodenough and Cozon [55]	84	45	Left pretibial area	Left groin	Not available
Sano et al. [56]	41	7	Dorsal nevus	Peritoneal seeding, omentum	Not available
Mansour and Kejariwal [57]	73	38	Neck	Brain, lungs	Not available
Otsu et al. [58]	70	11	Sole	Limb	Breslow depth 3 mm, Clark level III
Toba et al. [59]	46	10	Left sole	Lung-pulmonary nodule	Not available

## Data Availability

All data are included in the manuscript.
